# Reconsidering the spectral distribution of light: Do people perceive watts or photons?

**DOI:** 10.1177/14771535241246060

**Published:** 2024-05-01

**Authors:** C Martinsons, F Behar-Cohen, T Bergen, P Blattner, M Herf, C Gronfier, K Houser, S Jost, M Nilsson Tengelin, G Obein, L Schlangen, L Simonot, M Spitschan, A Torriglia, J Zeitzer

**Affiliations:** a Centre Scientifique et Technique du Bâtiment, Saint Martin d’Hères, France; b Centre de Recherche des Cordeliers, INSERM UMRS1138, Université Paris Cité, Sorbonne Université, Paris, France; c Assistance Publique - Hôpitaux de Paris, Hôpital Cochin, Ophtalmopôle, Paris, France; d Hôpital Foch, Suresnes, France; e Australian Photometry and Radiometry Laboratory, Melbourne, VIC, Australia; f Federal Institute of Metrology METAS, Bern-Wabern, Switzerland; g F.lux Software LLC, Los Angeles, CA, USA; h Centre de Recherche en Neurosciences de Lyon, INSERM U1028, CNRS UMR5292, Université de Lyon, Lyon, France; i Oregon State University, Corvallis, OR, USA; j ENTPE, Ecole Centrale de Lyon, LTDS, CNRS UMR5513, Vaulx-en-Velin, France; k RISE Research Institutes of Sweden, Borås, Sweden; l Laboratoire National de Métrologie et d’Essais, Paris, France; m Eindhoven University of Technology, Eindhoven, The Netherlands; n Institut Pprime, CNRS UPR3346, Université de Poitiers, Chasseneuil Futuroscope, France; o Technical University of Munich, Munich, Germany; p Max Planck Institute for Biological Cybernetics, Tübingen, Germany; q Center for Sleep and Circadian Sciences, Stanford University, Stanford, CA, USA

## Abstract

The spectral distribution is a fundamental property of non-monochromatic optical radiation. It is commonly used in research and practical applications when studying how light interacts with matter and living organisms, including humans. In the field of lighting, mis-conceptions about the spectral distribution of light are responsible for unfounded claims, which pervade the scientific and technical communities. Starting from the definition of the spectral distribution, this paper describes the ambiguities and errors associated with a purely graphical analysis of the spectral distribution. It also emphasizes the importance of considering the particle nature of light in research involving both visual and non-visual effects, which implies using the spectral distribution expressed in the photon system of units, a system that has been seldom used in lighting research for historical reasons. The authors encourage lighting engineers and researchers to determine which system is best suited to their work and then proceed with the correct use of spectral distributions and of spectral weighting functions for applications involving optical radiation.

## 1 Introduction

The spectral distribution is a property used to describe the optical radiation from a non-monochromatic source. It is a measure of the contribution of each component of optical radiation within a given spectral range. When applied to light and lighting, the spectral distribution is usually defined in terms of a radiometric quantity and reported as a function of wavelength. For instance, the spectral power distribution (SPD) of a light source is typically expressed as the spectral radiant flux in W nm^−1^ or the spectral irradiance (W m^−2^ nm^−1^) measured at a given distance and direction from the source.

The spectral distribution of light can be formulated as a function of other variables, such as the frequency or the wavenumber. Transforming the spectral distribution from one variable to another is not a simple change of variable or scale. This operation leads to several paradoxes that illustrate some common misconceptions about the spectral distribution of light. These misconceptions pervade many areas of research involving the biological effects of light, whether the investigated outcomes are visual or non-visual. Using the spectral distribution of a typical, commonly used white light-emitting diode (LED), this paper explains how the interpretation of the spectral distribution of light can easily be misleading or even go wrong.

The spectral distribution of light can also be described in terms of photon numbers, or quanta. Although this representation is well established in photochemistry and photobiology, it is seldom used in lighting research. Using the photon as the ‘unit of light’ imposes another choice of spectral distribution, that is, an alternative to the spectral distribution based on the radiometric system. Changing from one system to the other also affects certain spectral functions used in the assessment of light exposure, whereas other types of spectral functions are invariant to the system used.

Although the radiometric and photon-based approaches are both valid, they are each more suited for studying and describing different types of light-induced effects, as will be discussed in the last section of this paper.

## 2 Definition of the spectral distribution of light

The spectral distribution of light is defined in the International Lighting Vocabulary^1^ of the Commission Internationale de l’Éclairage (CIE) as being the density of a radiant or luminous or photon quantity *X* (*λ*) with respect to the wavelength *λ*. The spectral distribution is given by *X*_*λ*_ = *dX* (*λ*) / d*λ*.

In the field of photometry and radiometry, the wavelength is the most widely used variable to describe spectral distributions. However, spectro-scopists favour the use of the wavenumber ***σ*** (the inverse of the wavelength), whereas atomic physicists prefer using the photon frequency *ν* (in Hz) or the photon energy *hν*, usually expressed in eV. Therefore, the quantity *X* can be expressed as a function of other variables, giving corresponding spectral distributions, such as *X*_*ν*_, *X*_*σ*_, etc., in which case the expressions of units change accordingly.

The choice of measuring instrument may inform which quantity is directly available to describe the spectral distribution of light. For example, a diffraction grating spectrometer splits light by wavelength to its output angle. In Fourier-transform infrared spectroscopy, the use of the wavenumber 1/*λ* is directly linked to the Fourier transform applied to the measured interferograms. Optical radiations with very long wavelengths such as terahertz radiations can be detected using frequency-mixing schemes, leading to the choice of the frequency to describe spectral distributions in this range.

## 3 The influence of choosing the independent variable

The CIE definition of the spectral distribution of light does not explicitly specify how to perform the change of variables needed to express the spectral distribution as a function of another unit.

Being a density distribution function, this is not a simple substitution of variables. It obeys the conservation of the energy contained in the differential bandwidths of interest. For instance, in the case of changing the SPD from the unit of wavelength to the unit of frequency, the energy conservation implies the equality as given by Equation (1).

(1) $$\left|X_{\lambda }\text{d}\lambda \right|=\left|X_{\nu }\text{d}\nu \right|$$

where *X*_*λ*_ is the optical power in the differential wavelength bandwidth d*λ*, and *X*_*ν*_ is the optical power in the differential frequency bandwidth d*ν*. Since *ν* = *c*_m_/*λ* and d*λ*/d*ν* =− *λ*^2^/*c*_m_, where *ν* is the frequency, *λ* is the wavelength and *c*_m_ is the speed of light in the considered medium (usually standard air), the spectral distribution in frequency units is given by Equation (2):

(2) $$X_{\nu }=X_{\lambda }\lambda^{2}/c_{\text{m}}$$

Equation (2) shows that the change of unit is more than a change of variable. This transformation changes the shape of the distribution, thereby modifying the position and magnitude of the minima and maxima of the spectral distribution. This phenomenon is very noticeable with broad spectral distributions. Several authors^2,3^ observed that the peak of the spectral solar irradiance is near 500 nm (a visible wavelength) when plotted as a function of wavelength, and near 880 nm (an infrared wavelength) when plotted as a function of the frequency, challenging the common belief that human vision evolved to reach an optimum sensitivity where solar radiation is maximum.^3^

Unlike the spectral distribution of light, many other categories of spectral functions are not density distributions: spectral transmittance and reflectance functions, spectral responsivity functions and action spectra, such as the spectral luminous efficiency function for photopic vision,^4^ the human *α*-opic spectral sensitivities^5^ and wildlife photopigment responses.^6,7^ When considering the same system of assessment, for instance the radiometric system based on the optical power in watt, these spectral quantities can be expressed as functions of another independent variable by a simple variable change, for instance from wavelength to frequency, without altering the locations of their peaks and troughs. This does not always hold when changing the system of evaluation, as shown in the next section of the paper for a transition from the radiometric system to the photon system.

**Figure 1:**
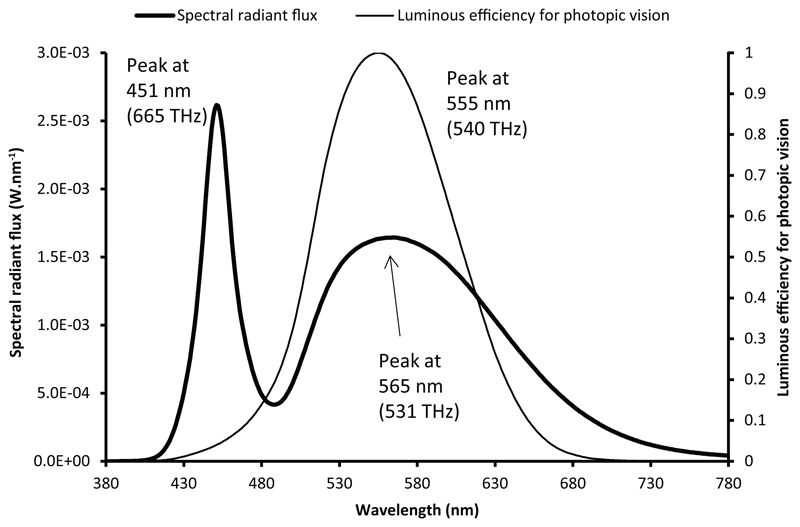
Spectral radiant flux of CIE LED B4 and luminous efficiency for photopic vision, plotted as a function of wavelength (radiometric system)

**Figure 2:**
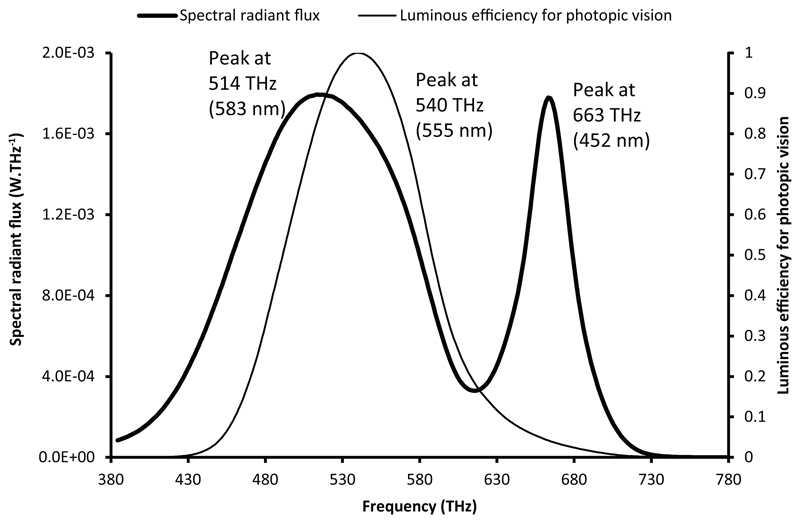
Spectral radiant flux of CIE LED B4 and luminous efficiency for photopic vision, plotted as a function of frequency (radiometric system)

When plotting together a spectral distribution and a spectral function, the change of independent variable creates an apparent mismatch between the two curves. To illustrate this phenomenon in the field of lighting, the spectral distribution of a typical phosphor-converted white LED, CIE LED B4, is studied. This spectral distribution was published by the CIE in a dataset of relative SPDs of common white LEDs.^8^ The shape of the spectral distribution exhibits a narrow blue peak and a broader peak corresponding to the fluorescent emission from phosphors. The data were vertically scaled to give a SPD corresponding to a radiant flux of 100 lm. The SPD as a function of frequency was computed using Equation (2). Figures 1 and 2 show the SPD plotted, respectively, as a function of wavelength (nm) and as a function of frequency (THz, or 10^12^ Hz). The two figures also show the spectral luminous efficiency function for photopic vision.

Although the peak position of the luminous efficiency for photopic vision does not change according to the chosen independent variable (555 nm or 540 THz), the relative heights and positions of the minima and maxima of the spectral distribution change between Figures 1 and 2. The change is more significant for longer wavelengths, as illustrated by a shift of the phosphor emission peak from 565 nm in the wavelength representation to 583 nm in the frequency representation, that is, a shift of 18 nm, whereas the shift of the blue emission peak is only 1 nm. The relative magnitude of the two peaks of this spectral distribution also changes between the two representations. In the wavelength plot, the peak corresponding to the blue emission of the LED is about 1.8 times higher than the phosphor emission peak. In the frequency plot, the phosphor peak and the blue peak have approximately the same magnitude. Similarly, the ‘width’ of each portion of the curve changes, so that lower peaks have a greater width, spread out in a way that preserves the area, while peaks that become higher tend to narrow the curve, again preserving total area.

These two representations are both correct. It is therefore not possible to determine the wavelength or the frequency of the ‘maximum emission’ using the spectral distribution. Assertions such as ‘the blue emission is more intense than the phosphor emission’ are not always justified. Furthermore, all the parameters derived from the magnitudes and the positions of the various peaks and troughs in the spectral distribution are ambiguous and may be misleading. For instance, this is the case of the ‘blue-to-yellow’ ratio, the ‘green spectral gap’ and other loosely defined characteristics of LEDs. In the field of integrative lighting and human-centric lighting,^9^ products are often advertised by purportedly showing a good match between the spectral distribution of light and a certain action spectrum.^10^ This type of data visualization is incomplete and may be misleading. The *y*-axis unit often being unspecified for the spectral distribution, this representation is inappropriate to illustrate the efficiency of light to produce the action of interest.

Reporting of wavelengths or frequencies of maximal or minimal emission should always be accompanied by clear specification of both axes of the spectral distribution. However, since the area under the curve is pertinent, reporting wavelengths or frequencies of maximal or minimal emission should be avoided unless also accompanied by a spectral plot that enables computation of the area under the curve. For distributions that are approximately Gaussian, it may be appropriate to report the peak wavelength and full width at half-maximum (FWHM).

Interpolating from the exclusive reporting of wavelengths or frequencies of maximal and minimal emission to derive photobiological or physiological properties should be avoided. The efficiency of light with respect to a given action spectrum is defined by an integral calculation.^11^ Because of the equality of Equation (1), an integral featuring the spectral distribution in the argument gives the same result, whatever the choice of unit. Integral parameters are therefore unambiguous. Such integral parameters are defined in several CIE technical notes,^12^ technical reports and international standards.^5^ Examples of parameters resulting from integrals over the spectral range include the luminous efficacy of radiation, the melanopic efficacy of luminous radiation, the blue-light hazard efficacy of luminous radiation^13^ and many others.

## 4 Considering the particle nature of light

Another type of distortion can be observed when converting a spectral distribution of light measured in the radiometric system to the corresponding spectral distribution of photons derived from their ‘count number’.^14^ In the photon system, the notion of radiant flux (in W) is replaced by the notion of photon flux^15^ expressed as the number of photons per second *N* with the unit of s^−1^.

Like the radiometric spectral distribution, the spectral photon distribution can be expressed as a function of the wavelength (*N*_*λ*_), or as a function of another variable such as the frequency (*N*_*ν*_). Each photon carries an energy *hν, h* being the Planck’s constant. Therefore, it is possible to express the spectral photon distribution, in wavelength units and in frequency units, from the radiometric spectral distributions using Equations (3) and (4):

(3) $$N_{\lambda }=X_{\lambda }\lambda /\left(hc_{\text{m}}\right)$$

(4) $$N_{\nu }=X_{\nu }/h\nu =X_{\lambda }\lambda^{3}/\left(hc_{\text{m}}^{2}\right)$$

**Figure 3:**
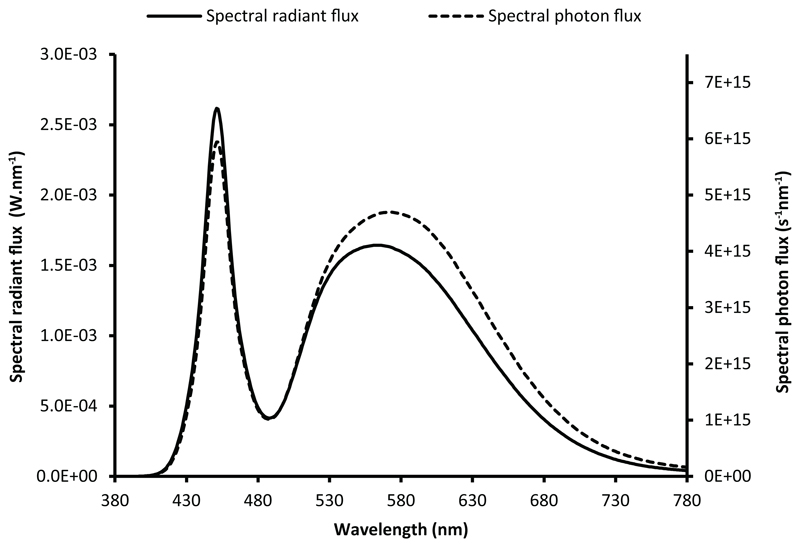
Spectral radiant flux of CIE LED B4 and spectral photon flux, plotted as a function of wavelength

Equations (3) and (4) show that the shapes of the spectral photon distribution and the SPD are different and depend on whether they are expressed in wavelength units or in frequency units. This can be explained by the fact that a photon of short wavelength (higher frequency) carries more energy than a photon of longer wavelength (shorter frequency). The resulting change in shape is illustrated in Figure 3, where the spectral radiant flux distribution and the spectral photon flux distribution of the LED described above are plotted on the same graph. The locations and relative magnitudes of the peaks and troughs of the spectral photon distribution are different from those of the SPD.

**Figure 4:**
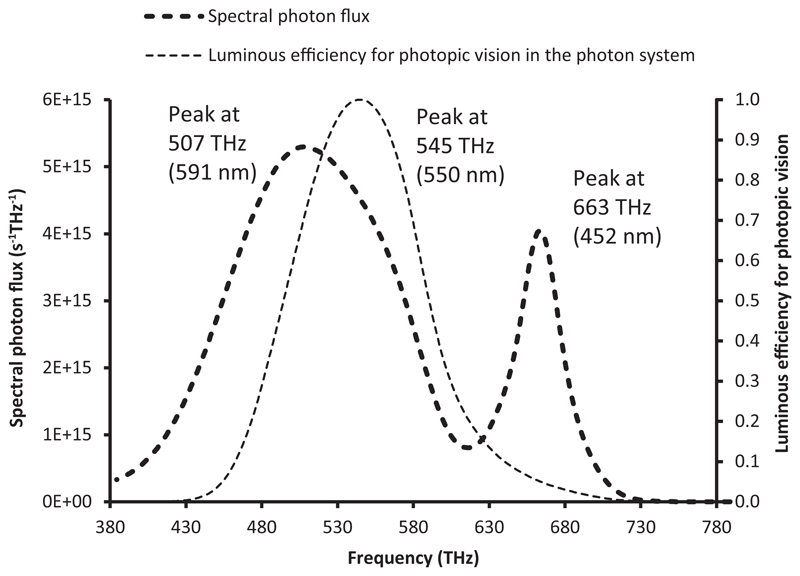
Spectral photon flux of CIE LED B4 and luminous efficiency for photopic vision, plotted as a function of frequency (photon system)

Figure 4 shows the spectral photon distribution of the chosen white LED, plotted as a function of frequency. The peak of the phosphor emission is located at 507 THz, which corresponds to a wavelength of 591 nm, about 26 nm away from the value determined with the spectral radiant flux plotted as a function of wavelength (565 nm). In addition, the phosphor emission peak is now higher than the blue peak (about 1.3 times), thereby confirming that the interpretation of the relative ‘strengths’ of the various wavelengths can be ambiguous and depends on what quantities are plotted on the *x*- and *y*-axes of the chosen spectral distribution.

This change in shape has more profound implications than the impact of choosing one independent variable or another. When using the spectral photon distribution, the spectral weighting functions must also relate to photon numbers, and not to radiometric quantities.

The Bureau International des Poids et Mesures (BIPM) gives specific guidelines in Appendix 3 of the ninth edition of the SI brochure^14^ for transforming action spectra from one system to the other. These guidelines are applicable to photobiological and photochemical action spectra as well as to spectral sensitivity functions used in vision sciences (cone-fundamental spectral sensitivities, luminous efficiencies for photopic, scotopic and mesopic vision, *α*-opic spectral sensitivities, etc.).

Following the BIPM guidelines, an action spectrum should be expressed by explicitly specifying the system (radiometric or photon-based) that was used to establish them. If another system is used, then the action spectrum should be scaled using the inverse relationship of Equation (3). Using the notations of the BIPM, a general response process ‘*A*’ can be described by the spectral weighting function *S*_*p*, *A*_ (*λ*) in the photon system and by *S*_*e*, *A*_ (*λ*) in the radiometric system. The relationship between the two functions is given by Equation (5)^14^:

(5) $$S_{p,A}(\lambda )=\gamma_{A}\frac{hc}{\lambda \cdot n_{a}(\lambda )}S_{e,A}(\lambda )$$

where *γ*_*A*_ is a constant that normalizes the maximum values of *S*_*p*, *A*_ (*λ*) to 1, *h* is the Planck’s constant, *c* is the speed of light in vacuum and *n*_*a*_ (*λ*) is the refractive index of standard air as a function of wavelength.

**Figure 5:**
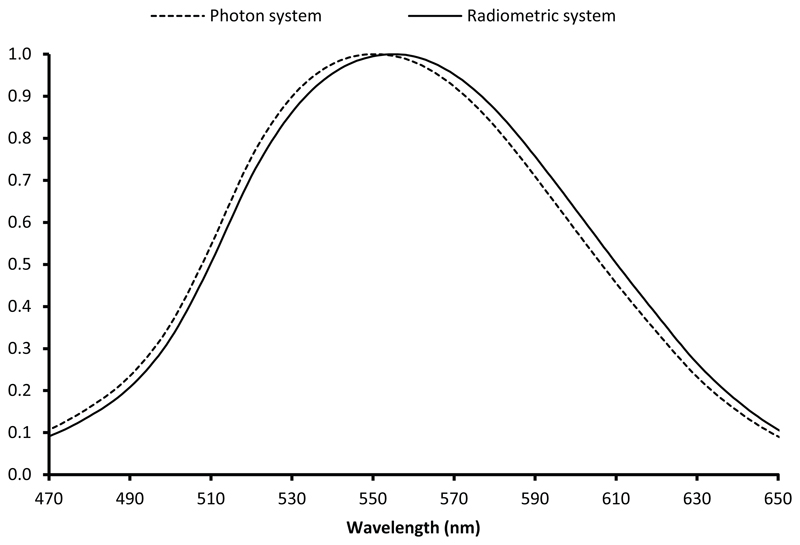
Spectral luminous efficiency function for photopic vision in the radiometric system and in the photon system. The FWHM is the same in the two systems

The application of Equation (5) to the luminous efficiency function for photopic vision gives the expression for the photon-based equivalent function of Equation (6):

(6) $$V_{p}(\lambda )=\frac{\lambda_{p}}{\lambda }V(\lambda )$$

where *λ*_*p*_ ≈ 552.7915 nm. This photon-based function peaks at 550 nm (545 THz), as compared with 555 nm (540 THz) for its equivalent in the radiometric system, as shown in Figure 5. The *V*_*p*_ (*ν*) curve in the photon system is represented as a function of frequency in Figure 4.

The application of Equation (5) to the standardized melanopic spectral weighting function^5^ underlying many non-visual effects of light^16–24^ yields a photon-based function with a peak at 488 nm (614 THz) while this function peaks at 490 nm (612 THz) in the radiometric system.

The BIPM guidelines can also be applied to the spectral responsivity of optical detectors.^25^ By using the transformation of Equation (5), the quantum efficiency function can be derived. These two spectral functions are different, but they measure the same effect in two different systems of units (radiometric system for the spectral responsivity and photon system for the quantum efficiency).

Unlike action spectra and spectral responsivity functions, the spectral transmittance, spectral reflectance and spectral absorbance have the same expressions in the radiometric and in the photon-based system. At any given wavelength, the value of such functions is a ratio of power (i.e. transmitted power vs. incident power), which is exactly equal to the ratio of numbers of photons (i.e. number of transmitted photons vs. number of incident photons) of the same wavelength. This is the case, for instance, with the spectral transmittance of the eye.^26^ When considering the total transmittance (or reflectance or absorbance) across a spectral range of interest, the values are different between the two systems. For example, the total transmittance in terms of power is different from the total transmittance in terms of number of photons.

## 5 Choosing the photon or the radiometric system?

The commonly used description of the spectral distribution of light in terms of radiometric quantities expressed as a function of wavelength (spectral radiant flux, spectral irradiance, etc.) may be explained by the fact that the CIE has popularized this representation since 1924, when the spectral luminous efficiency function *V* (*λ*) for photopic vision was standardized.^27^ At the time, quantum physics was rapidly progressing, but the ‘quantum of light’ was not yet considered as being a particle. The widespread acceptance of the concept of the ‘photon’ started in 1926,^28^ about 3 years after the famous experiment of Arthur Compton,^29^ which firmly established the photon as a particle carrying both energy and momentum. The role of the photon in human vision was demonstrated more than 30 years later, when George Wald discovered in 1958 that incident photons change the configuration of the rhodopsin molecule thereby initiating the transduction of visual signals into nerve impulses.^30^ William Rushton strengthened this finding by introducing in 1970 the principle of univariance^31^ stating, in his original terms, that ‘the output of a receptor depends upon its quantum catch, but not upon what quanta are caught’.

In fact, most photobiological phenomena implied in visual and non-visual light detection by animals, including humans, involve photochemical reactions between photons and complex organic molecules. In 1985, Richard Mansfield found that the spectral sensitivities of primate photopigments have a common shape when expressed in the photon system of units and plotted as a function of the photon frequency.^32^ Several other mathematical formulas were later established^33^ and found to be applicable to humans^34^ (rods and cones) and to other mammalian species.^35^ This finding showed that when using the photon system, the spectral sensitivity functions of the different mammalian visual pigments have a common template peaking at a specific frequency characterizing each visual pigment.

Despite the role of the photon in photobiology and photochemistry, the lighting community prominently uses the spectral distribution of light expressed with radiometric quantities to study and predict the visual and non-visual effects of light. Characterizing the amount of ‘active light’ consists in weighting the spectral distribution by action spectra, such as the *α*-opic spectral sensitivities,^5^ the luminous efficiency for photopic vision *V*(*λ*) or the blue-light hazard function *B* (*λ*), which is the action spectrum defined by the International Commission on Non-Ionizing Radiation Protection^13^ for blue-light-induced photoretinopathy. This operation gives meaningful efficacy and efficiency figures, as well as equivalent photometric quantities, such as the *α*-opic equivalent daylight illuminances,^5,11^ provided that the considered action spectrum is expressed in the correct system according to the guidelines of BIPM.^14^ It should be noted that two action spectra describing the same effect in two different systems have a different shape and, that the peak wavelength of the effect is different when expressed in photon quantities or radiometric quantities.

It is important to emphasize that both systems of evaluation are perfectly correct. However, they are related to two different aspects of the optical radiation. The radiometric calculation is based on the optical power, whereas the photon assessment considers the quantum nature of light: a flow of photons each carrying a quantum of energy *hν*. The radiometric system is intrinsically better suited to describe thermal phenomena, whereas photochemical reactions are better described in terms of photons.

Indeed, the field of actinometry employs chemical dosimeters that undergo light-induced reactions, where measurement of the reaction rate enables the calculation of the absorbed photon flux.^36^ This is also the reason why the quantities used to measure light contributing to photosynthesis, a complex biological process involving photochemical reactions, are based on photon units. The photosynthetic photon flux is determined using a photon-based spectral weighting function applied to the spectral photon distribution. This quantity is measured in micromoles of photons per second.

The best practices for reporting light exposure in laboratory experiments and field settings with human participants^37–41^ include measuring the spectral distribution of the stimulus from the observer’s point of view. Since the visual and non-visual effects mediated by retinal mechanisms involve photochemical reactions in photoreceptors, they are dependent on the number of captured photons within a specific range of energies *hν* or frequencies *ν*.

There is burgeoning interest in the non-image forming impacts of light (circadian rhythms, sleep, metabolism, mood, etc.). Many biological studies now investigate these impacts using quantitative methods based on measuring melatonin,^22^ collecting physiological signals,^42^ etc. *In vitro* studies of photobiological processes are also carried out using a range of molecular biology tools such as the Terminal deoxynucleotidyl transferase dUTP Nick End Labelling (TUNEL) technique used to investigate DNA fragmentation in the retinal cells after exposure to blue light.^43^ In all these instances, designing experiments with light exposures controlled in terms of photon units can ensure that the same number of photons can be delivered in exposures of different spectral distributions. It is useful to notice that stimuli matched in photons are not necessarily matched in energy, and vice versa.

Experimental designs with exposures that are controlled in terms of photon units could help gain more robust and meaningful insights into dose–effect relationships and response thresholds, as illustrated in a study designed to measure irradiance–response curves and action spectra in the photon system for melatonin suppression and circadian resetting as a function of exposure duration.^44^ This approach could also help establish more defensible targets for outdoor lighting and architectural lighting when the potential biological impact of light is a concern.

The photon system can also be more appropriate in the study of processes triggered by low light levels (small photon counts), when the quantum nature of light can be predominant. An example of such study involving very low levels of light tested the sensitivity of human vision using single photons.^45^ In this study, low light imaging technologies such as single-photon avalanche diodes (SPADs) were used to detect individual photons emitted by a light source. SPAD arrays^46^ have been increasingly used for high-resolution single-photon imaging in very diverse fields such as quantum communication^47^ and fluorescence lifetime imaging^48^ of biological samples.

## 6 Conclusions

This paper first points out several misconceptions associated with an incorrect interpretation of the spectral distribution of light. The locations and magnitudes of the peaks and troughs in the spectrum of a light source may change significantly according to the choice of representation. With the white LED chosen as an example, the peak of the phosphor emission is at 565 nm in the traditional wavelength representation, but it is located at 583 nm in the frequency representation, and at 591 nm in the system of units based on the number of photons as a function of frequency. It is therefore not possible to define the ‘wavelength of maximum emission’ without ambiguity. The shape of the spectral distribution is significantly affected by the choice of the plotting variable and by the choice between a radio-metric or a photon-based assessment. The relative magnitude of the peaks and troughs in the distribution greatly differs with the chosen representation. For instance, a typical white LED spectral distribution reaches its maximum value in the blue range in the radiometric assessment using a wavelength representation, whereas its maximum value lies in the yellow range when using the photon system of units and a frequency representation. Therefore, loosely defined metrics, such as the blue-to-yellow ratio, can be misleading, and their use should be avoided. A greater attention should be paid by the lighting community when plotting together spectral distributions and spectral weighting functions such as sensitivity curves or action spectra. Such graphs are sometimes employed by lighting manufacturers to illustrate that their products emit a light that matches a chosen sensitivity curve, a visualization that can only hold in a specified system of evaluation and when using consistent units.

A second point was addressed by this paper. When considering its particle nature, light is best described by using the spectral photon distribution. With the photon system of units, the spectral distribution significantly differs from the commonly used SPD. The apparent distortion in the shape of the spectral photon distribution is a result of the energy of a single photon being inversely proportional to the wavelength.

When changing from one assessment system to the other, the spectral weighting functions that are applied to the spectral distribution will also change. This is the case for the action spectra commonly used in photochemistry, photobiology and lighting: when an action spectrum is expressed in the photon system, its shape and peak wavelength are different as compared to the radiometric system. However, this is not the case for the spectral transmittance, reflectance and absorbance: these remain invariant between the two systems. The consistency of the system of units between the spectral distribution and the action spectra is necessary to ensure the correct assessment of efficacy figures, such as the luminous efficacy of radiation and *α*-opic equivalent daylight quantities.

When research experiments are aimed to study the interaction of light with matter or living organisms, the choice of working with photon units or radiometric units should be determined by the nature of the effects induced by light. Photothermal effects are intrinsically better described by radiometric quantities. Photochemical and photobiological effects, including vision and non-visual effects mediated by the eye, intrinsically involve the action of photons, justifying the choice of the photon system in experimental designs and light exposure assessments. Dose–effect relationships in the photon system of units may provide a more detailed and physiologically relevant insight into visual and non-visual (non-image forming) effects. When considering phenomena happening in dark or dim light conditions, the radiometric system is not well adapted because the incident energy may be so small that only a few photons may be caught by photoreceptors.

The use of more ecologically relevant instruments such as wearable and portable sensors to measure light exposures combined with highly controlled experimental conditions and increased accuracy in measuring biological and physiological outcomes means that the choice between the two types of light dosimetry, radiometric or photon-based, may have noticeable consequences in the interpretation of experimental data. In any case, it is important to state which system is used when reporting data and applying spectral weighting functions.

## Supplementary Material

Supplementary Material
